# Phase-separation antagonists potently inhibit transcription and broadly increase nucleosome density

**DOI:** 10.1016/j.jbc.2022.102365

**Published:** 2022-08-11

**Authors:** Rajyalakshmi Meduri, Linda S. Rubio, Suman Mohajan, David S. Gross

**Affiliations:** Department of Biochemistry and Molecular Biology, Louisiana State University Health Sciences Center, Shreveport, Louisiana, USA

**Keywords:** hexanediol, transcriptional condensates, phase separation, chromatin, Heat Shock Factor 1, Msn2/Msn4, *Heat Shock Protein* gene coalescence, RNA Pol II, RNA Pol III, budding yeast, 3C, chromosome conformation capture, cDNA, complementary DNA, ChIP, chromatin immunoprecipitation, GSTF, gene-specific transcription factor, HD, hexanediol, HS, heat shock, Hsf1, Heat Shock Factor 1, HSP, heat shock protein, NHS, non–heat shock, qPCR, quantitative PCR, SDC, synthetic dextrose complete, SE, super-enhancer, YPDA, yeast extract–peptone–dextrose with adenine

## Abstract

Biomolecular condensates are self-organized membraneless bodies involved in many critical cellular activities, including ribosome biogenesis, protein synthesis, and gene transcription. Aliphatic alcohols are commonly used to study biomolecular condensates, but their effects on transcription are unclear. Here, we explore the impact of the aliphatic dialcohol, 1,6-hexanediol (1,6-HD), on Pol II transcription and nucleosome occupancy in budding yeast. As expected, 1,6-HD, a reagent effective in disrupting biomolecular condensates, strongly suppressed the thermal stress–induced transcription of Heat Shock Factor 1–regulated genes that have previously been shown to physically interact and coalesce into intranuclear condensates. Surprisingly, the isomeric dialcohol, 2,5-HD, typically used as a negative control, abrogated Heat Shock Factor 1–target gene transcription under the same conditions. Each reagent also abolished the transcription of genes that do not detectably coalesce, including Msn2/Msn4-regulated heat-inducible genes and constitutively expressed housekeeping genes. Thus, at elevated temperature (39 °C), HDs potently inhibit the transcription of disparate genes and as demonstrated by chromatin immunoprecipitation do so by abolishing occupancy of RNA polymerase in chromatin. Concurrently, histone H3 density increased at least twofold within all gene coding and regulatory regions examined, including quiescent euchromatic loci, silent heterochromatic loci, and Pol III-transcribed loci. Our results offer a caveat for the use of HDs in studying the role of condensates in transcriptional control and provide evidence that exposure to these reagents elicits a widespread increase in nucleosome density and a concomitant loss of both Pol II and Pol III transcription.

Biomolecular condensates are self-organized membraneless intracellular structures that lack a fixed stoichiometry and are stabilized by weak, primarily hydrophobic, multivalent interactions between proteins or between proteins and either RNA or DNA. They can form under physiological conditions (*e.g.*, nucleolus, nuclear pore complex, Balbiani bodies) or in response to stress (*e.g.*, P bodies, stress granules). Such structures play important roles in regulating various cellular processes, including RNA metabolism, protein synthesis, ribosome biogenesis, DNA damage repair, and cell type-specific gene transcription, thereby enabling cellular differentiation or permitting cells to evade stressful environments (1, 2). Condensates have been observed in both prokaryotes (3, 4) and eukaryotes (reviewed in (5)), underscoring their critical evolutionarily conserved roles. In the nucleus, condensation of various regulatory factors has been implicated in gene control. These include gene-specific transcription factor (GSTF)-, coactivator-, and Pol II-containing condensates that assemble at super-enhancers (SEs) (6, 7, 8, 9, 10); splicing factors that condense into Cajal bodies, nuclear speckles, and paraspeckles (11); and the RNA Pol II C-terminal domain that forms recruitment-, initiation-, elongation-, and splicing-specific condensates (12, 13).

Electrostatic, weak hydrophobic, pi–pi stacking, and pi–cation interactions are critical in the formation of most biomolecular condensates (5). A common approach to demonstrating the phase-separated and liquid droplet–like nature of these intracellular structures is to expose cells to the aliphatic alcohol 1,6-hexanediol (1,6-HD). Such treatment rapidly melts membraneless bodies such as Cajal bodies and stress granules (14). It also disrupts transcriptional condensates at SE-associated genes; accompanying this is loss of the coactivators BRD4 and MED1 as well as RNA Pol II (10). Similar treatment with the chemically related 2,5-HD or 1,4-butanediol, each less hydrophobic than 1,6-HD, typically has little or no effect (14). These are thus commonly employed as negative controls. For example, exposure of MCF7 breast cancer cells to 1,6-HD but neither 2,5-HD nor 1,4-butanediol disrupted coactivator complexes assembled at estradiol-regulated SEs, as well as the transcription associated with them (9). Table 1 summarizes recent studies of nuclear condensates that have employed these reagents.

**Table 1:** Use of dialcohols in the study of nuclear condensates

Study	Alcohol	Model organism/system	Concentration and time frame used	Assays	Key outcomes	Reference
1	1,6-HD, 2,5-HD, 1,4-butanediol (1,4-BD), and 1,5-pentanediol (1,5-PD)	*In vitro* (cell free)	5, 10, and 15% for 0–20 h	*In vitro* droplet and light-scattering assays	FUS hydrogel, hnRNPA2 liquid-like droplets, and intermediate filaments dissolved in a time-dependent manner following treatment with 1,6-HD and to a lesser extent with 1,5-PD, but not with either 2,5-HD or 1,4-BD	(14)
		HeLa cells	6–8% for 5 min	Immunofluorescence (IF)	Cajal bodies, nuclear speckles, and stress granules were dissolved following treatment with 1,6-HD or 1,5-PD but not with either 2,5-HD or 1,4-BD	
2	1,6-HD	*Drosophila* S2 and mouse NIH3T3 cells	10% for 2 min	Microscopy	1,6-HD treatment resulted in significant dispersal of HP1 from heterochromatic domains but did not change histone enrichment	(47)
3	1,6-HD	*In vitro*	10%	Droplet assay	1,6-HD treatment reduced BRD4-IDR and MED1-IDR droplet opacity	(10)
		mESCs	3% for 15 s for microscopy; 1.5% for 30 min for ChIP	IF and ChIP-Seq	Disruption of MED1 and BRD4 condensates by 1,6-HD was accompanied by a loss of chromatin-bound MED1 and BRD4 at SEs as well as a loss of RNA Pol II at SEs and SE-driven genes. Occupancy of all three proteins was also reduced at typical enhancers although the effect was greater at SEs	
4	1,6-HD	mESCs	10% for 3–10 min	Live-cell imaging by lattice light-sheet microscopy	Dissolution of Mediator and Pol II condensates in a time-dependent manner	(7)
5	1,6-HD and 2,5-HD	U2OS cells	2, 5, and 10% for 0–90 s	Confocal fluorescence microscopy; lattice light-sheet microscopy; FRAP	2% and 5% 1,6-HD dissolved Sp1 LCD (low-complexity domain)-containing hubs (puncta) in live cells; FUS LCD-containing hubs were also dissolved, although less rapidly. 2,5-HD melts self-aggregated LCD hubs more slowly and less thoroughly than 1,6-HD at the same concentration	(8)
6	1,6-HD and 2,5-HD	HAP1 cells	6–10% for 5 min	RNA FISH, IF, and super-resolution microscopy	Paraspeckles with colocalized NEAT and NONO disappeared with 1,6-HD but not with 2,5-HD treatment	(48)
7	1,6-HD and 2,5-HD	*In vitro*	5–10%	Liquid droplet assay	Pol II C-terminal domain (CTD) (human and yeast) liquid droplets were equally sensitive to 1,6-HD and 2,5-HD	(12)
8	1,6-HD	*In vitro* and HeLa cells	2.5% and 10%	*In vitro* droplet assay and IF	Cyclin T1-IDR droplets were sensitive to 10% 1,6-HD. 2.5% 1,6-HD disrupted histidine-rich domain (HRD)–mediated phase separation but not direct HRD–CTD binding, thereby preventing P-TEFb-mediated CTD hyperphosphorylation	(49)
9	1,6-HD, 2,5-HD, and 1,4-BD	MCF7 cells	7.5 to 8.5% HD was used for 5 min either pre- or post-17β-estradiol (E_2_) treatment	RT–qPCR, GRO-Seq, and ChIP-Seq	1,6-HD strongly limited E_2_-induced RNA expression from MegaTrans enhancers and Estrogen Receptor alpha (ERα)-target genes, whereas little or no effect was observed in cells comparably treated with either 2,5-HD or 1,4-BD. ERα was still effectively recruited to MegaTrans enhancers following 1,6-HD treatment, yet assembly of other MegaTrans components (GATA3 and AP2γ) was disrupted	(9)
10	1,6-HD	mESCs	10% for 17 min	Live-cell imaging	1,6-HD dissolved SRSF2 condensates representing splicing factories/nuclear speckles	(13)
11	1,6-HD	Fission yeast	10% for 3 min	Single-molecule FISH	Sme2 lncRNA–Smp protein complexes exhibit phase separation properties that are sensitive to 1,6-HD. Such treatment disrupts the pairing of associated loci during meiosis	(50)
12	1,6-HD	U2OS cells	1% for 30 min	Time-lapse live-cell microscopy	53BP1 DNA damage response foci were sensitive to 1,6-HD, impairing DNA repair efficacy	(51)
13	1,6-HD	SEM leukemia cells	1.5% for 30 min	ChIP-Seq and nascent RNA-Seq	Global loss of BRD4 and Mediator at typical enhancers, although most significantly at SEs. eRNA and transcription of SE-target genes was affected. Enhancer–promoter looping was not affected	(52)
14	1,6-HD	Purified recombinant proteins and budding yeast cells	5% or 10% for ≤60 s (*in vitro*); 5% for 10 min (*in vivo*)	Reconstitution of cohesin rings on lambda DNA; ChIP	1,6-HD rapidly disrupts cohesin–DNA condensates *in vitro*. Treatment of yeast cells with 5% 1,6-HD for 10 min disrupts cohesin–DNA condensates based on decreased Smc1 ChIP signal at intergenic regions	(53)
15	1,6-HD	NIH3T3 and U2OS cells	10% for 1 min, IF; 0.5% for ≤10 min, live-cell imaging; 0.5% and 10% for 15–120 min, RT–qPCR	IF, live-cell imaging, and RT–qPCR	The Q-rich IDR of Med15 fused to a short hydrophobic motif was required to assist in condensate assembly, suggesting that hydrophobic residues could serve as adhesive elements to promote condensate formation. 0.5% 1,6-HD rapidly dissolved Med15 condensates. Pretreatment with 0.5%, unlike with 10%, had little or no effect on the kinetics of serum-induced immediate early gene (IEG) expression	(54)
16	1,6-HD	Recombinant proteins, complexes purified from Sf9 cells and HeLa cell lysates	0.625 to 10% for 15 min or 5% for 1 h	*In vitro* kinase, phosphatase, DNase, and polymerase assays	1.25% 1,6-HD strongly impaired kinases and phosphatases and partly inhibited DNA polymerases but had no effect on DNase activity. At 5–10% 1,6-HD, both kinases and phosphatases were completely inactivated	(55)
17	1,6-HD and 2,5-HD	Human cell lines (HeLa, RPE-1, HCT116, and DLD-1)	2.5%, 5%, or 10% for 5 min	Live-cell single-nucleosome imaging; photoactivated localization microscopy (PALM)	Both 1,6-HD and 2,5-HD immobilize chromatin motion and hypercondense chromatin in a concentration-dependent manner and appear to do so directly, rather than affecting chromatin through melting of condensates	(37)
18	1,6-HD	293 cells	10% for 1 min	Confocal fluorescence microscopy; ChIP-Seq	NUP98–HOX9, a chimeric oncoprotein linked to leukemogenesis, formed phase-separated assemblies (puncta) in live cells; 1,6-HD dissolved these puncta and drastically reduced NUP98–HOX9 genome-wide binding	(41)
19	1,6-HD and 2,5-HD	mESCs	1.5% for 2 min	Structured illumination microscopy (SIM); live-cell single nucleosome imaging; BAT-Hi-C	Exposure of mESCs to 5% and 10% (but not 1.5%) 1,6-HD for 2 min resulted in chromatin hypercondensation, nuclei shrinkage, formation of abnormal protein aggregates, and cell death. Exposure of mESCs to 1.5% 1,6-HD for 2–5 min reduced the number of BRD4, MED1, and Pol II puncta associated with active transcription; puncta associated with constitutive heterochromatin (HP1α, H3K9me3); nuclear speckles (SC35); polycomb bodies (Ring1B); nucleoli (nucleolin); and CTCF clusters. 1.5% 2,5-HD treatment for 2 min largely dissolved BRD4 and MED1 condensates. Other condensates were unaffected, and cells retained viability. BAT Hi-C: 1–5 min 1.5% 1,6-HD treatment decreased long-range interactions, increased short-range interactions, and strengthened compartmentalization, yet had no effect on enhancer–promoter interactions	(40)
20	1,6-HD	Human colorectal cancer cell lines	1% for 6 h	Fixed cell microscopy; dual DNA/RNA FISH	1% 1,6-HD treatment for 6 h at 37 °C did not affect clustering of cMyc-containing ecDNA circles but strongly inhibited cMyc transcription	(56)
21	1,6-HD	HeLa	10% for 1 min or 1.5% for 1 h (both at 42 °C)	Stochastic optical reconstruction microscopy (STORM)	10% 1,6-HD for 1 min dissolved heat shock–induced HSF1 droplets and markedly diminished HSF1 occupancy of chromatin (yet had no effect on HSF1–DNA interactions *in vitro*). 1.5% 1,6-HD for 1 h suppressed HS-induced RNA levels	(42)

FRAP, fluorescence recovery after photobleaching; mESC, mouse embryonic stem cell.

In the budding yeast *Saccharomyces cerevisiae*, *Heat* *Shock* *Protein* (*HSP*) genes regulated by Heat Shock Factor 1 (Hsf1) are transcriptionally activated and engage in robust physical interactions in response to acute heat shock (HS), coalescing into intranuclear foci (15, 16). Such foci may reflect the formation of biomolecular condensates (17, 18). Interestingly, genes regulated by the alternative heat-responsive transcription factors, Msn2/Msn4 (hereafter referred to as Msn2), while also strongly induced (19), do not engage in prominent physical contact as assayed by both chromosome conformation capture (3C) and fluorescence microscopy. Likewise, constitutively expressed genes do not exhibit 3C interactions (15, 16). Thermally induced Hsf1-dependent genes are occupied by large quantities of Hsf1, Pol II, and the coregulators Mediator, SAGA, Swi/Snf, and Rpd3 (20, 21, 22, 23, 24). Enrichment of the transcriptional machinery together with physical convergence of *cis*-regulatory elements and extensive chromosomal looping interactions bears resemblance to SEs (17). Similar to SEs, the clustering of HS-activated Hsf1-dependent genes is accompanied by exceptionally high transcriptional output, >10-fold higher than the typical Pol II gene (17).

Here, we have tested the hypothesis that perturbation of *HSP* gene foci by 1,6-HD negatively impacts their transcriptional output, in analogy to SE-regulated mammalian genes (9, 10). While we found that 1,6-HD abrogated *HSP* gene transcription as expected, surprisingly 2,5-HD had an equally repressive effect. Moreover, both HDs abolished transcription of heat-induced Msn2-regulated genes, and this effect extended to constitutively regulated housekeeping genes as well as Pol III-transcribed genes. Chromatin immunoprecipitation (ChIP) analysis revealed that while activator recruitment was only mildly affected, RNA polymerase occupancy within promoter and gene bodies was abolished. These results demonstrate that at moderately elevated temperatures, aliphatic dialcohols potently affect the recruitment of RNA polymerase and/or prevent its retention on DNA. In parallel, histone H3 occupancy was significantly increased, well beyond the histone density typically seen under transcriptionally quiescent conditions.

## Results

### Both 1,6-HD and 2,5-HD potently inhibit Hsf1-dependent transcription

**Figure 1:**
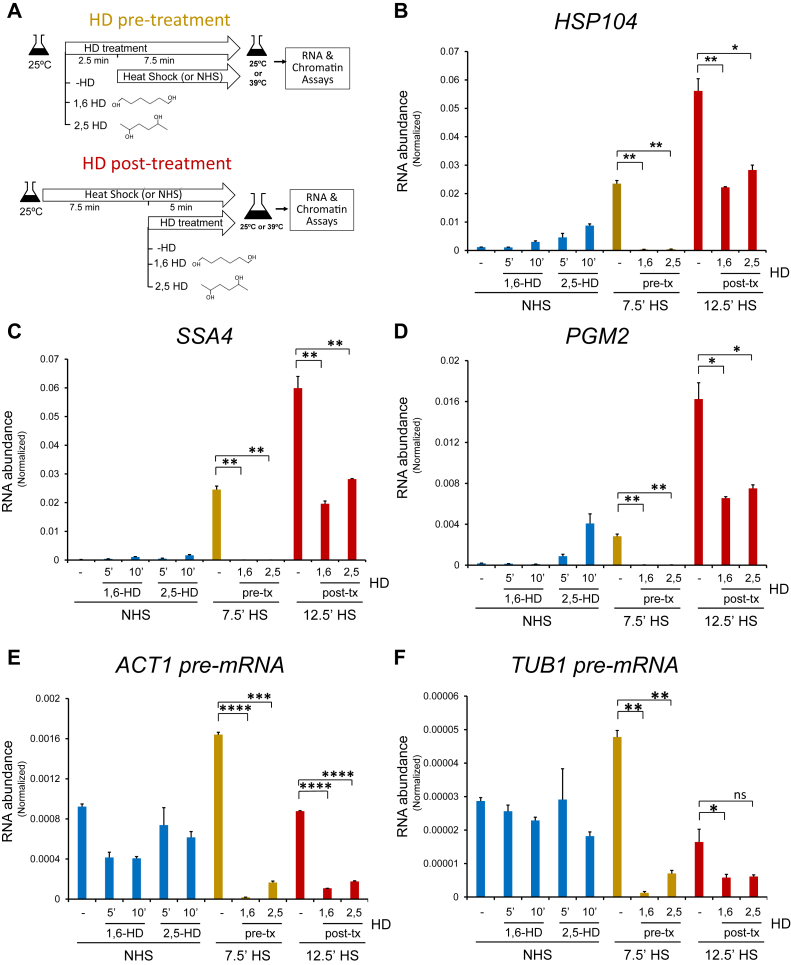
**Phase separation antagonists potently inhibit transcription of both heat shock (HS)–inducible and constitutively expressed genes.***A*, experimental strategy used in hexanediol (HD) pretreatment and post-treatment protocols. In HD pretreatment, cells were treated for 2.5 min with 5% 1,6-HD or 2,5-HD (or neither [−HD]) followed by 7.5 min HS at 39 °C or continued incubation at 25 °C (non–heat shock [NHS]). In HD post-treatment, cells were subjected to 7.5 min heat shock (or not) followed by exposure to 5% 1,6-HD or 2,5-HD for 5 min. HS was terminated by adding 20 mM sodium azide for the RNA abundance assay or 1% formaldehyde for ChIP. *B*–*F*, RNA abundance assays. RNA was isolated from W303-1A cells subjected to pre-HD or post-HD treatment (pre-tx and post-tx, respectively) as indicated, and the abundance of individual mRNAs, measured by RT–qPCR, was normalized to that of the Pol III transcript *SCR1*. *B* and *C*, Hsf1-dependent genes. *D*, Msn2/Msn4-dependent gene. *E* and *F*, constitutively regulated genes. For *ACT1* and *TUB1*, primers were designed to span the exon–intron junction to permit measurement of pre-mRNA levels; their low abundance is consistent with their transient presence. Error bars represent standard deviation of 2 independent biological replicates (qPCR = 4). Significance was calculated using the two-tailed *t* test (∗*p* < 0.05; ∗∗*p* <0.01; ∗∗∗*p* <0.001; and ∗∗∗∗*p* <0.0001; ns, not significant). ChIP, chromatin immunoprecipitation; qPCR, quantitative PCR.

As discussed previously, HS-activated Hsf1-dependent genes coalesce into prominent intergenic clusters that may reflect formation of transcriptional condensates (17, 18). Therefore, if condensate formation is important for transcriptional output, the expression of Hsf1-target genes in cells treated with 1,6-HD—a potent dissolver of biomolecular condensates—will be diminished, perhaps abolished. In contrast, Msn2 targets, which do not detectably coalesce (16), should be less affected. In addition, 2,5-HD, which has little or no effect on condensates, would be anticipated to have less impact on Hsf1-induced transcription in analogy to previous observations of estrogen receptor–regulated SEs (9). To test these hypotheses, we used two different strategies (summarized in Fig. 1*A*). In the first, “HD pretreatment,” cells were pretreated with 5% 1,6-HD or 2,5-HD for 2.5 min at 25 °C followed by upshift to 39 °C over the next 7.5 min. In the second, “HD post-treatment,” cells were subjected to a 25 to 39 °C upshift for 7.5 min followed by addition of 5% HD over the final 5 min. In both protocols, transcription was terminated by addition of 20 mM sodium azide, RNA was isolated, and transcript abundance was measured by RT–quantitative PCR (qPCR). The concentration of HD and times of incubation were selected based on previous work with mammalian nuclear condensates (Table 1).

In control (HD-untreated) cells, transcript levels of the Hsf1-dependent genes *HSP104*, *SSA4*, and *HSP82* were strongly and progressively induced by a 25 to 39 °C upshift for 7.5 to 12.5 min (Figs. 1, *B* and *C* and S1*A*; −HD samples, compare *blue*, *gold*, and *red bars*), consistent with previous observations (24). In contrast, when cells were pretreated with either 1,6-HD or 2,5-HD and then heat shocked, there was virtually no detectable transcript (Figs. 1, *B* and *C* and S1*A*; 7.5 min HS samples). In the post-treatment protocol, when cells were heat shocked for 7.5 min followed by HD treatment for the final 5 min (12.5 min HS total), heightened transcript levels were observed for the three genes, yet transcript abundance was frozen at 7.5 min heat-shocked levels (Figs. 1, *B* and *C* and S1*A*), consistent with a normal transcriptional response for the first 7.5 min. Collectively, these results argue that HS-induced transcription is abrogated upon exposure of cells to either 1,6-HD or 2,5-HD. Interestingly, in cells exposed to 2,5-HD and maintained at the non–heat shock (NHS) temperature of 25 °C, a modest increase in transcript abundance was observed in certain cases (Fig. 1*B* and S1*A* [*blue bars*]; Figure S4), suggesting that 2,5-HD can modestly stimulate the transcription of select genes.

**Figure 2:**
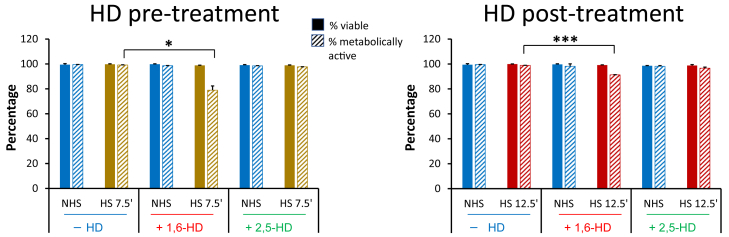
**Cells retain short-term viability and metabolic activity following either the pre- or post-hexanediol (HD) protocol.** Bar graphs depict the percentage of viable and metabolically active cells (assayed by trypan blue staining and methylene blue dye exclusion, respectively) immediately following either pre-HD or post-HD treatment. Following HD/heat shock (HS) treatment, cells were stained with trypan blue for 1 min and methylene blue for 5 min; ∼400 cells were screened within each biological replicate (N = 2). Non–heat shock (NHS), 25 °C; HS, 42 °C. *Solid bars*, trypan blue; *striped bars*, methylene blue. Error bars represent standard deviation of 2 independent biological replicates. The *t* test was conducted as for Figure 1. ∗*p* < 0.05; ∗∗∗*p* <0.001.

One explanation for the dramatic cessation of transcription is that 5% HD combined with moderately elevated temperature is deleterious to global metabolic activity such that even short-term cell viability is severely compromised. To rule this out, we measured cell viability by trypan blue staining and metabolic status by methylene blue dye exclusion in cells treated (or not) with HD. By these criteria, cells maintained at 25 °C were viable and metabolically active following either 5 min or 10 min treatment with 5% 1,6-HD or 2,5-HD, similar to untreated cells (Fig. 2, NHS samples). Under HS conditions, HD-untreated cells were 100% viable and metabolically active. Likewise, cells exposed to HD, either before or after HS, largely retained viability and metabolic activity (Fig. 2, HS samples). Hence, cells retain short-term viability when exposed to low concentrations of either 1,6-HD or 2,5-HD coupled with brief HS.

### HDs inhibit transcription of noncoalescing Msn2-dependent genes as well as that of constitutively expressed genes

Next, we asked whether HD treatment affected the transcription of Msn2-regulated genes that previous work has shown do not physically cluster upon their activation by HS (16). Similar to its effect on Hsf1-dependent genes, HD pretreatment obviated transcription of *PGM2* or *CTT1*, whereas HD post-treatment froze 12.5 min heat-shocked cells at 7.5 min levels (Fig. 1*D* and S2*B*). Therefore, the repressive effect of HDs extends to HS-induced genes that do not prominently coalesce. A similar outcome was observed for the dual Hsf1- and Msn2-regulated *HSP12* gene (Fig. S1*C*) that has been shown to coalesce (15, 16).

To address whether HDs affect constitutive Pol II transcription, we designed primers spanning exon–intron junctions to specifically identify nascent pre-mRNA transcripts. Using this strategy, we found that nascent transcript abundance of both *ACT1* and *TUB1* was strongly suppressed using either the HD pretreatment or HD post-treatment protocol (Fig. 1, *E* and *F*). Consistent with this, mature transcript levels of these genes were also reduced (data not shown). Thus, at moderately elevated temperature, 1,6-HD and 2,5-HD inhibit not only the transcription of HS-induced genes but also that of housekeeping genes.

### Aliphatic dialcohols inhibit Pol II recruitment to both HS-induced and constitutively regulated genes

We next investigated the mechanism by which HD exposure, in concert with elevated temperature, inhibits Pol II transcription. A number of scenarios could be imagined. In one, the GSTF is prevented from gaining access to its cognate binding site (UAS) in chromatin. In another, the GSTF gains access, but recruitment of RNA polymerase is prevented. In a third, both GSTF and Pol II recruitment are permitted, but subsequent steps in the transcriptional cascade—namely, promoter escape and elongation—are prevented.

**Figure 3:**
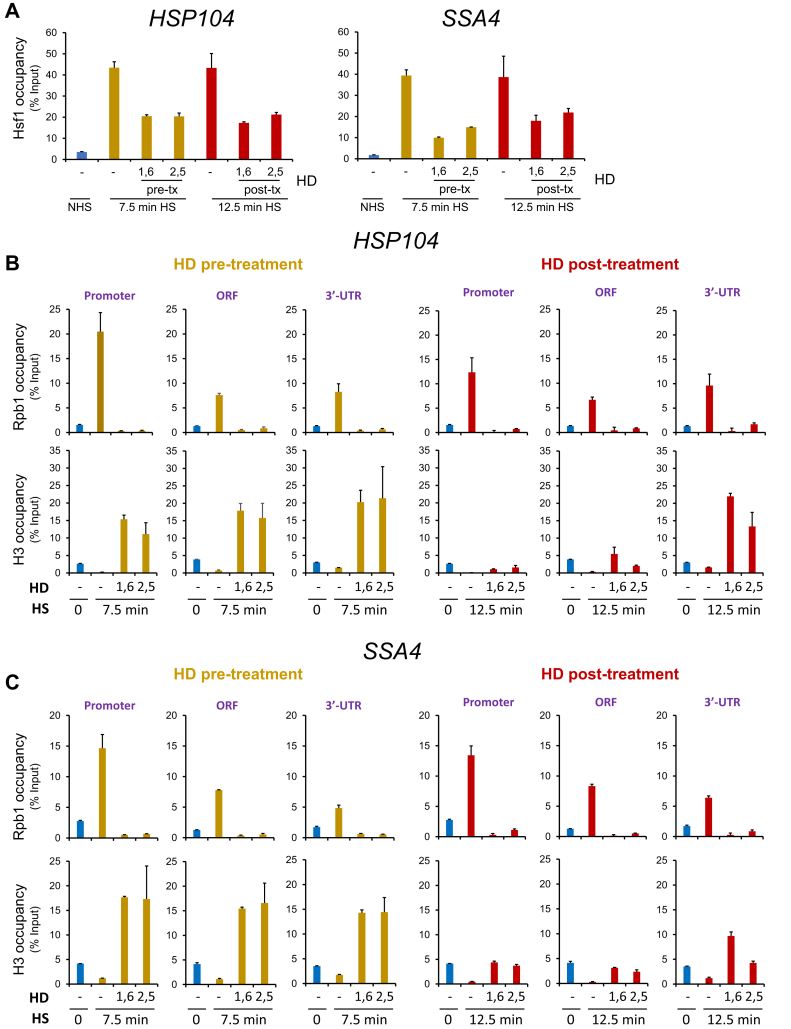
**Aliphatic dialcohols in combination with elevated temperature elicit a dramatic increase in nucleosome density that is permissive to Hsf1 binding yet refractory toPol II occupancy at Hsf1-dependent genes.***A*, Hsf1 occupancy of *HSP104* and *SSA4* in W303-1A cells subjected to either pre-HD or post-HD treatment followed by crosslinking with 1% HCHO. Chromatin immunoprecipitation (ChIP) was conducted as described in Experimental procedures. *B* and *C*, Pol II (Rpb1) and histone H3 occupancy of promoter, ORF, and 3′-UTR of *HSP104* and *SSA4* in cells subjected to either pre-HD or post-HD treatment followed by ChIP as above. Error bars represent the standard deviation of 2 independent biological replicates (quantitative PCR = 4). HD, hexanediol; Hsf1, Hsf1, heat shock factor 1.

To distinguish between these possibilities, we performed ChIP, initially addressing the effect of HDs on Hsf1 recruitment to *HSP* genes. Under NHS conditions, Hsf1 is present in the nucleus in an inactive complex with Hsp70 and the Hsp70 cochaperone Sis1 (25, 26, 27, 28). Following exposure to thermal stress, nascent proteins misfold that titrate Hsp70 (29, 30), triggering Hsf1 binding to chromatin (31, 32). Treatment of cells with either 1,6-HD or 2,5-HD did not prevent HS-induced Hsf1 binding at *HSP104* or *SSA4*, although it did reduce its occupancy compared with the HD-untreated control (Fig. 3*A*). At *HSP82*, 1,6-HD treatment prevented the further increase in Hsf1 binding that occurs in response to HS (Fig. S2*A*). As this enhanced occupancy has been previously attributed to cooperative binding of Hsf1 to low-affinity HS elements (33), the ability of 1,6-HD to antagonize weak hydrophobic interactions may contribute to this.

**Figure 4:**
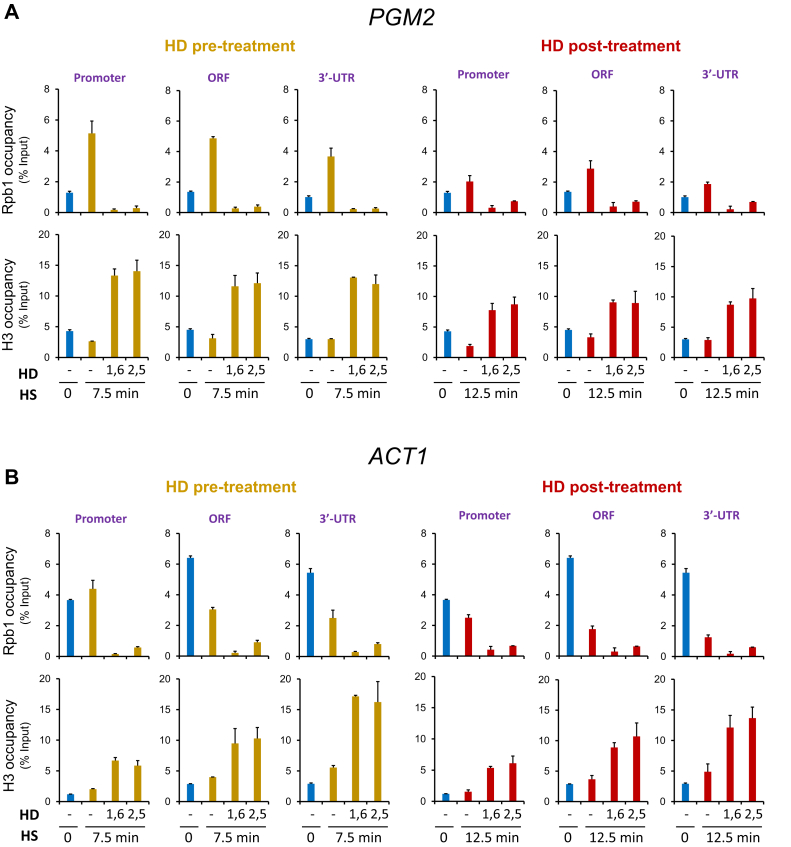
**Aliphatic dialcohols dramatically increase nucleosome occupancy and inhibitPol II recruitment at Msn2-regulated as well as at constitutively transcribed genes.***A* and *B*, Pol II and H3 chromatin immunoprecipitation (ChIP) analysis of *PGM2* and *ACT1* in cells subjected to either pre-HD or post-HD treatment was conducted as described in Fig. 3. HD, hexanediol; HS, heat shock.

In contrast to the mild effects of HD treatment on Hsf1 occupancy in heat-shocked cells, pre-HD or post-HD treatment abolished Pol II occupancy of its target genes within both regulatory and coding regions (Figs. 3, *B*and *C* and S2B). Similar results were obtained for Msn2- and dual-regulated genes (Figs. 4*A* and S3*A* and *B*). These observations are therefore consistent with the notion that HD abrogates thermal stress–induced gene transcription whether it is added prior to or during exposure to elevated temperature. Analysis of the constitutively expressed *ACT1* and *TUB1* genes led to a similar outcome (Figs. 4*B* and S3C). Notably, such suppression went well beyond the reduction in Pol II recruitment and nascent transcription that typically occurs in response to thermal stress at housekeeping genes (34, 35). Collectively, the results indicate that loss of Pol II recruitment and/or Pol II retention within chromatin underlies the cessation of transcription.

### HDs dramatically increase nucleosome occupancy at both thermally induced and constitutively expressed genes as well as at Pol III-transcribed genes

To gain further insight into the role played by HDs, we examined H3 occupancy, which serves as a proxy for nucleosome density. As previously observed, acute HS induces gene-wide displacement of histones at Hsf1 target genes (Fig. 3, *B* and *C* and S2*B*) (20, 22). Pretreatment with either 1,6-HD or 2,5-HD not only prevented this remodeling, it led to a significant increase in H3 occupancy over both regulatory and coding regions (Fig. 3, *B* and *C* and S2*B*). This increased nucleosome density, as much as 15-fold over the HD-untreated basal state, offers a potential mechanism to account for the decrease in Pol II occupancy elicited by the HDs. The increase in nucleosome density implied by these ChIP data is all the more remarkable in light of the minimal transcription occurring at these genes under NHS conditions (Fig. 1, *B* and *C* and S1*A*) (32). The increase in H3 abundance is not typically seen in cells post-treated with HD, particularly at those *HSP* gene loci (promoter, ORF) where histones have already been displaced by prior HS (Fig. 3, *B* and *C* and S2*B*; compare *red bars* with *gold*). At genes whose chromatin is less dynamically regulated—*PGM2*, *HSP12*, *ACT1*, and *TUB1*—both pre-HD and post-HD treatment led to a substantial enhancement of H3 density (twofold to fivefold) throughout regulatory and coding regions (Figs. 4 and S3, *B* and *C*). These results indicate that the combination of elevated temperature and HD stabilize chromatin structure to a remarkable degree, in certain cases exceeding by 10-fold the nucleosome compaction seen in control noninduced cells.

**Figure 5:**
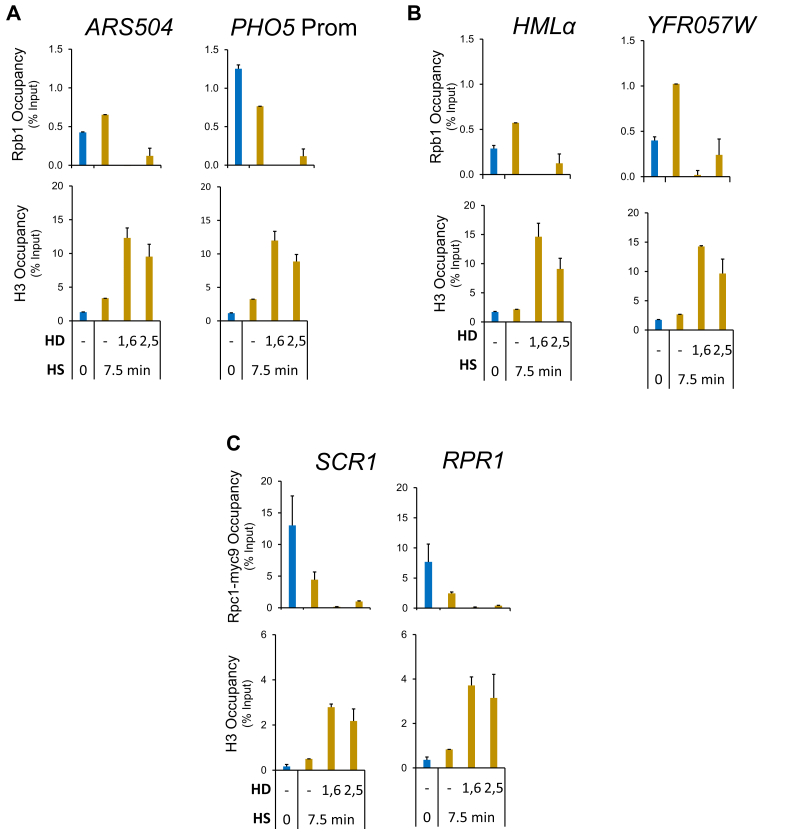
**DualHD/HS treatment increases nucleosome density while obviating RNA polymerase recruitment at both euchromatic and heterochromaticPol II genes as well as at transcriptionally activePol III genes.***A* and *B*, Pol II and H3 chromatin immunoprecipitation (ChIP) analysis of the indicated Pol II loci in cells subjected to the HD pretreatment protocol as described in Fig. 1. *A*, inactive euchromatic loci. Prom, promoter. *B*, *HMLα* and *YFR057w* (ORF regions) assembled in *SIR*-mediated heterochromatin. *C*, Pol III (Rpc1-myc9) and H3 ChIP analysis of *SCR1* and *RPR1* (coding regions). HD, hexanediol; HS, heat shock.

**Figure 6:**
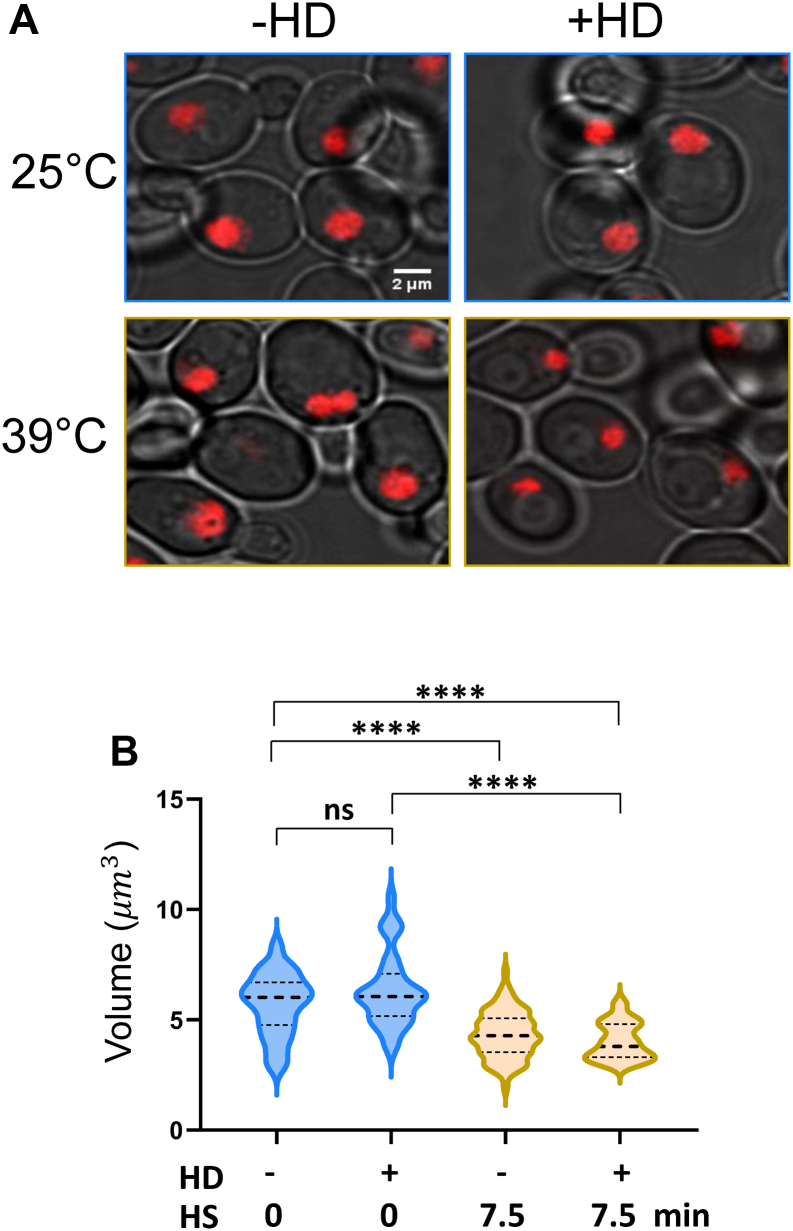
**Exposure of yeast cells to the hexanediol (HD)/heat shock (HS) protocol is accompanied by a significant reduction in chromatin volume.***A*, fluorescence micrographs of midlog DBY1447 diploid cells expressing H2A-mCherry maintained under non–heat shock (NHS) conditions (*top* L), exposed to HD alone (*top* R), exposed to HS alone (*bottom* L), or subjected to both (HD pretreatment protocol; *bottom* R). HS: 25 to 39 °C shift for 7.5 min. HD: 5% 2,5-HD exposure for 10 min total, with the first 2.5 min being at 25 °C. Images were acquired using an Olympus CSU W1 Spinning Disk Confocal Microscopy System. *B*, single-cell analysis of nuclear volumes. Midlog DBY1447 cells were treated, and images acquired, as in *A*. The volume occupied by H2A-mCherry in each cell was determined using the 3D Object Counter plugin in Fiji/ImageJ. Violin plots are quantifications of 35 to 210 individual cells. All *p* values were calculated using two-tailed Mann–Whitney *U* test (∗∗∗∗*p* <0.0001; ns, not significant [*p* = 0.26]).

To provide additional evidence for this striking phenomenon, we used three strategies. First, we investigated the effects of the HD pretreatment protocol on histone density and Pol II occupancy at weakly expressed euchromatic loci and *SIR*-silenced heterochromatic loci. As shown in Figure 5, *A* and *B*, quiescent (*ARS504* and the *PHO5* promoter) and heterochromatic (*HMLα* and *YFR057w*) loci responded in a similar fashion to HD/HS treatment as the transcriptionally active ones. Therefore, even at loci that are rarely transcribed, the combination of HD (especially 1,6-HD) and HS suppresses Pol II occupancy to near-undetectable levels while dramatically increasing histone H3 density. Second, we asked if HD pretreatment similarly impacted actively transcribing Pol III genes. Indeed, occupancy of Rpc1, the large Pol III subunit, is nearly obviated at two such genes, *SCR1* and *RPR1*; concomitantly, H3 occupancy at each increased ∼10-fold. Thus, the profound effect of HD/HS treatment on RNA polymerase and nucleosome occupancy is not restricted to Pol II transcribed loci. Third, we attempted to demonstrate one aspect of the phenomenon, increased chromatin compaction, using an orthogonal approach. For this analysis, we used an H2A-mCherry–expressing diploid yeast strain and determined chromatin volumes in cells exposed to HD (2,5-HD), HS, both HD and HS, or neither one. Consistent with the notion that chromatin compaction increased in cells exposed to the HD/HS treatment, the volume of chromatin decreased significantly (∼25%) in cells exposed to the dual treatment (Figure 6*A*, compare upper L *versus* lower R; Fig. 6*B*, compare −HD, −HS *versus* +HS, +HD). While exposure to 2,5-HD alone had little effect—consistent with H3 ChIP analysis of NHS cells (Fig. S4)—exposure to 7.5 min HS strongly diminished nuclear volume and to a similar degree (∼20%) to the HD/HS treatment (Fig. 6, *A* and *B*). The HS result is consistent with a recent report that yeast cells subjected to mild HS exhibit a diminished nuclear volume (36). Therefore, since HS alone typically increased nucleosome density only slightly (evident at non–stress-responsive genes [Figs. 4*B* and 5 and S3*C*]), diminished nuclear volume cannot be the entire basis for the substantial increase in nucleosome compaction arising from dual HD/HS exposure.

### Cells lose long-term viability following acute exposure to HDs and thermal stress

**Figure 7:**
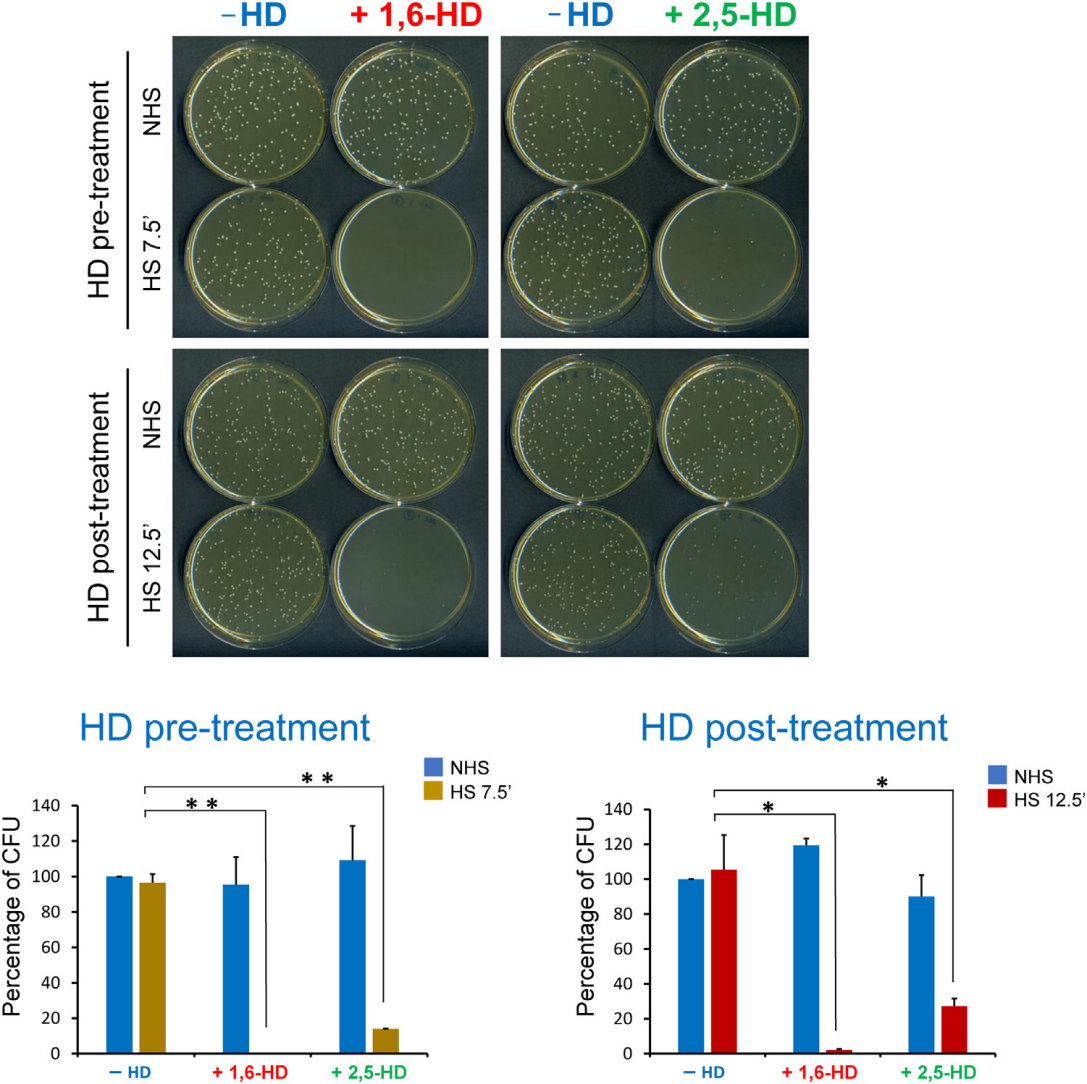
**Cells subjected to either the pre-HD or post-HD protocol lose long-term viability.***Top*, YPDA agar plates on which ∼300 cells, subjected to either pre-HD or post-HD treatment, were spread. Plates were incubated for 2 days at 30 °C prior to imaging. *Bottom*, bar graph summaries of 2 independent experiments. *t* Test was conducted, and *p* values represented as for Figure 1. CFU, colony-forming unit; HD, hexanediol; YPDA, yeast extract–peptone–dextrose with adenine.

Since transcription of both Pol II and Pol III genes was abolished as a consequence of the HD/HS protocol, we wondered if long-term vitality of the cells was affected. We measured viability by the capacity of the cells, with or without HD treatment, to form colonies when grown on rich medium at an optimal temperature (30 °C). Cells were able to form colonies following either 5- or 10-min exposure to 5% HD at 25 °C and to a degree similar to that observed for untreated cells (Fig. 7, *blue bars*). Likewise, cells subjected to thermal stress alone (either 7.5 or 12.5 min) were able to fully recover (Fig. 7, −HD bars). However, when cells were simultaneously exposed to HS and HD (either pre- or post-treatment protocol), their ability to form colonies was severely compromised (Fig. 7, +1,6-HD and +2,5-HD). The results suggest that the loss of transcription of stress-responsive, housekeeping, and structural RNA genes is associated with long-term loss of viability. Notably, as discussed previously, short-term viability was relatively unaffected (Fig. 2). Therefore, even though cells were viable and metabolically active during the time frame of the experiment, they lost their capability to transcribe the crucial genes required for their long-term survival.

## Discussion

Here, we demonstrate that when yeast cells are exposed to either 1,6-HD or 2,5-HD in combination with an elevated, but physiologically relevant temperature, Pol II transcription is rapidly and quantitatively suppressed. Genes whose transcription is associated with intergenic clusters/condensates are strongly impacted but so too are genes that do not detectably coalesce and whose transcription is not as prominently linked to condensates. It is therefore probable that the impact on transcription stems from perturbation of something other than, or in addition to, biomolecular condensates. We propose that a key determinant driving the loss of transcription is HD/heat-induced chromatin compaction. Our reasoning is as follows.

First, we find that H3 ChIP signals are two-fold to 15-fold enhanced within both regulatory and coding regions of all genes analyzed in HD/HS-treated cells. This applies to not only actively expressing Pol II genes but also to transcriptionally quiescent and silent heterochromatic genes. It even applies to Pol III genes. Together, these observations imply that the combination of HD and HS elicits a widespread, possibly genome-wide, increase in nucleosome density, a density that is likely sufficient to impair transcription. It is notable that exposure to HDs alone (*i.e.*, under NHS conditions [25 °C]) does not have the same effect: 2,5-HD causes a slight increase in Pol II occupancy, whereas it has no effect on H3 density; 1,6-HD has no effect on Pol II occupancy although it does slightly increase H3 abundance at certain genes (Fig. S4). Second, we observed that the HD/HS protocol resulted in ∼25% reduction in chromatin volume as determined by fluorescence microscopy of an H2A-mCherry–expressing diploid. However, as a similar reduction was observed in cells subjected to HS alone, reduced nuclear volume is unlikely to be the only mechanism underlying increased nucleosome density. Third, Maeshima *et al.* have shown through single-nucleosome tracking that both 1,6-HD and 2,5-HD immobilize HeLa cell chromatin under conditions very similar to those used in the present study—namely, 2.5, 5, or 10% HD at 37 °C for 5 min (37). Using photoactivated localization microscopy, the authors in addition observed that increasing concentrations of 1,6-HD beyond 5% elicited an increase in nucleosome clustering in the same cells. Collectively, the results of these studies suggest that in evolutionarily diverse cell types (budding yeast, HeLa) 1,6-HD and 2,5-HD elicit hypercondensation of chromatin when cells are maintained at temperatures between 37 and 39 °C.

The alternative scenario, that chromatin compaction occurs secondarily to transcription inhibition induced by HD/HS, is less likely. In this scenario, the relatively low abundance of H3 at weakly expressed genes (such as *SSA4* and *HSP104* in NHS cells) is due to their low—but nonzero—level of transcription that might result in H3 displacement and potential unfolding of higher-order chromatin structure. However, the fact that the HD/HS protocol elicits a substantial increase in H3 abundance at genes embedded in silent heterochromatin (Fig. 5*B*) whose transcription is virtually undetectable (38) argues against this. Also arguing against this idea is the fact that 1,6-HD has been observed to promote cation-dependent nucleosome condensation *in vitro* (37), a phenomenon independent of RNA polymerase. These arguments notwithstanding, our data do not rule out that the two events—HD/HS-mediated inhibition of RNA polymerase and HD/HS-mediated compaction of nucleosomal arrays—are concerted.

There are several intriguing implications to our findings. First, the UAS regions of *HSP* genes tend to remain partially accessible to Hsf1 under conditions in which their promoter and coding regions are rendered inaccessible to Pol II. Constitutive presence of nucleosome-antagonizing factors within these genes’ upstream regions (32) may foster Hsf1 access. Second, given the absence of Pol II ChIP signal within gene-coding regions, it is possible that a final round of Pol II elongation is permitted following addition of either HD. Alternatively (or additionally), Pol II may dissociate from chromatin under such conditions. Third, the significant increase in ChIP signal we observe in cells subjected to the HD/HS protocol may represent 3D compaction of tetrasomes (39), consistent with the aforementioned observations of increased chromatin compaction in mammalian cells (37, 40).

The results presented here provide a caveat to recent studies that have used HDs in the study of nuclear condensates (summarized in Table 1). Studies that have employed 1,6-HD without the use of a suitable negative control might be subject to alternative interpretations. For example, Wang *et al.* (41) used 10% 1,6-HD to demonstrate the oncogenic role of transcriptional condensates containing the NUP98–HOXA9 chimeric protein in a leukemic cell line. While the authors reported that 1,6-HD disrupted NUP98–HOXA9 condensates and diminished DNA binding and transcriptional potency of the chimeric transcription factor, enhanced compaction of chromatin might have contributed to this. Likewise, Sun *et al.* (42) reported that human HSF1 forms phase-separated condensates in response to HS and that such condensates, which are enriched for transcriptional coactivators, dissolve when cells are briefly exposed to 10% 1,6-HD. Concomitantly, HS-induced transcription is abolished genome-wide. While these observations support the interpretation that formation of HSF1-containing condensates is required for HS-induced transcriptional activation, it is possible that such treatment also results in chromatin compaction. This effect on higher-order chromatin structure may play a role in suppressing HSF1-mediated transcription in human cells as we have observed with budding yeast.

Finally, we note that the HS/HD protocol described here may have wider applicability. Typically, the conditional *rpb1-1* mutant (43) is used to interrogate the role of Pol II in phenomena such as chromatin occupancy of a transcription-associated protein. This approach is not ideal as Pol II occupancy is only moderately (twofold to threefold) reduced at actively transcribing genes following a 90 min shift to the nonpermissive temperature (37 °C) (*e.g.*, (44)). By contrast, HD pretreatment followed by a 7.5 min shift to 39 °C, as described here, results in close to 100% loss of Pol II occupancy at both constitutively and inducibly transcribed genes.

In conclusion, while others have pointed out limitations and caveats to the use of 1,6-HD in studying biomolecular condensates (14, 45), our study is the first to demonstrate that brief exposure to either 1,6-HD or 2,5-HD at a physiologically relevant temperature elicits a near-quantitative block in transcription. This is accompanied by loss of RNA polymerase within both regulatory and coding regions and a concomitant dramatic increase in H3 abundance. Our data indicate that both 1,6-HD and 2,5-HD induce high levels of nucleosome compaction. We suggest that it is this effect on tertiary chromatin structure that is primarily responsible for the cessation of transcription.

## Experimental procedures

### HS and HD treatment

Strains used in this study are listed in Table S1. In all molecular and viability assays, cells were grown to midlog (absorbance at 600 nm = 0.5–0.8) in YPDA medium (YPD [yeast extract–peptone–dextrose] supplemented with 0.002% adenine) at 25 °C. To elicit HS, equal volumes of culture and prewarmed YPDA medium (55 °C) were rapidly mixed by pouring into Erlenmeyer flasks mounted in a shaking water bath (Shel Lab) preheated to 39 °C. For HD treatment, one-ninth volume of freshly prepared 50% (w/v) 1,6-HD (Sigma–Aldrich; catalog no.: 240117; crystalline) or 50% (w/v) 2,5-HD (Sigma–Aldrich; catalog no.: H11904; liquid) solutions (both made up in YPDA) were added to cultures to achieve a final concentration of 5% HD (423 mM). For HD pretreatment, cells were incubated at 25 °C in YPDA containing no HD, 5% 1,6-HD or 2,5-HD for 2.5 min following which HS was performed at 39 °C for 7.5 min. For HD post-treatment, 39 °C HS was performed for 7.5 min followed by addition of one-ninth volume of YPDA containing no HD, 50% 1,6-HD, or 50% 2,5-HD for an additional 5 min. In all cases, NHS samples maintained at 25 °C in −HD, 5% 1,6-HD, or 5% 2,5-HD were used as controls.

### RNA isolation and mRNA quantification

To measure total RNA abundance, cells from a 12.5 ml midlog culture were metabolically poisoned by adding 20 mM sodium azide (final concentration) following HS/HD treatment, harvested at 4 °C, and lysed by vortexing with glass beads in 300 μl 200 mM Tris–HCl (pH 7.5), 500 mM NaCl, 10 mM EDTA for 10 min at 4 °C, twice with a break of 2 min on ice. Nucleic acids were then purified by phenol–chloroform extraction and precipitated through addition of 3 volumes of ice-cold 100% EtOH, 300 mM sodium acetate, and 2 μl of RNA-grade glycogen (Thermo Fisher Scientific; catalog no.: R0551), incubating on ice for 2 h. RNA was then pelleted by centrifugation, washed in 70% ethanol, air dried, and dissolved in 50 μl of nuclease-free water.

TURBO-DNase kit (Invitrogen; catalog no.: AM1907) was used to remove DNA contamination, and a 2 μg aliquot of purified RNA was used to synthesize complementary DNA (cDNA) (High-Capacity cDNA Reverse Transcription Kit; Thermo Fisher Scientific; catalog no.: 4368813) in a Bio-Rad thermal cycler set at 25 °C for 10 min, 37 °C for 2 h, and 85 °C for 5 min in a 60 μl reaction volume. Following this, 2 μl of the resultant cDNA were used for qPCR using Bio-Rad 2× iTaq Universal SYBR Green Supermix and gene-specific primers. Relative cDNA levels were calculated using the standard curve method (23). To correct for variation in the recovery of cDNA templates, PCR signal from the *SCR1* Pol III transcript was used as a normalization control. Primer sequences are provided in Table S2.

### ChIP

To perform ChIP assays, 50 ml midlog W303-1A cultures were used in either the HD pretreatment or HD post-treatment protocol. In each, HS was terminated by adding formaldehyde to a final effective concentration of 1% (363 mM). Given that HD reacts in a 1:1 stoichiometry with HCHO to form an acetal, to compensate for its presence, 786 mM (2.16%) formaldehyde was added to the +HD samples (5% HD = 423 mM). Following 10 min incubation at either 39 °C (HS samples) or 25 °C (NHS samples), 363 mM glycine was added for another 10 min to quench unreacted HCHO. Cells were harvested at 4 °C, washed, and stored at −80 °C. Cell lysis, chromatin sonication, and immunoprecipitation were conducted essentially as described (16). Specifically, from a total of 2000 μl chromatin lysate obtained from the above, 400 μl were used for each IP. Antibodies used were as follows: 1.5 μl anti-Hsf1 (polyclonal, rabbit; Gross laboratory), 1.5 μl anti-Rpb1 (polyclonal, rabbit; Gross laboratory; raised against mouse C-terminal domain), 1.0 μl anti-Histone H3 (polyclonal, rabbit; Abcam, catalog no.: ab1719), and 2.5 μl anti-Myc (9E10 monoclonal, mouse; Santa Cruz Biotechnology, catalog no.: sc-40). Immunoprecipitation was performed using preblocked Protein A Sepharose beads (Cytiva; catalog no.: GE17-0780-01) for 16 h at 4 °C. Washing and elution were conducted as previously described, and DNA was purified using phenol–chloroform–isoamyl alcohol extraction followed by ethanol precipitation. The DNA was dissolved in 60 μl of nuclease-free water. In parallel, input samples were processed to serve as normalization controls. About 2 μl of DNA were used in each qPCR, which was conducted and quantified as described previously. Primer sequences are provided in Table S2.

### Quantification of chromatin volume

DBY1447 diploid cells expressing H2A-mCherry (a gift of Donna and Jason Brickner) were grown to midlog (absorbance at 600 nm = 0.6) in synthetic dextrose complete (SDC) medium supplemented with adenine. Independent cultures were grown for each of three conditions (HS, HD, and HD/HS); a paired NHS control was evaluated in each case. Cells were attached to a smart substrate with reservoir (VA-HEAT, Interherence GmbH) by coating the substrate with 100 μg/ml concanavalin A (Sigma–Aldrich) prior to cell attachment. Cells were incubated for 20 min at 25 °C; the medium was then replaced with either SDC or SDC supplemented with 5% 2,5-HD for 2.5 min. Cells were then subjected to an instantaneous 7.5 min 25 to 39 °C HS by using the VA-HEAT temperature controller (or kept at 25 °C). Images were acquired using an Olympus CSU W1 Spinning Disk Confocal Microscopy System set at 10% laser power. For each field, 11 z-planes (each imaged for 100 ms) were taken with interplanar distance of 0.5 μm using a 100× objective. To quantify volume occupied by H2A-mCherry, we used the 3D Object Counter plugin in Fiji/ImageJ (Open-Source) (46). A manual threshold was applied to each image to remove background. Data were plotted using GraphPad Prism 8 (GraphPad Software, Inc).

### Viability and metabolic activity assays

To measure the viability and metabolic status of the cells immediately following HS/HD treatment (HS conducted at 42 °C), a 100 μl aliquot of midlog culture of RMY005 (absorbance at 600 nm = 0.5) was diluted 1:10 in 1 ml YPDA (25 °C). The cell suspension was centrifuged and resuspended in 30 μl YPDA to remove most traces of HD. For the viability assay, cells were stained with an equal volume of 0.4% trypan blue (Sigma; catalog no.: T6146) in phosphate-buffered saline for 1 min. To assay metabolic activity, cells were stained with 0.1 mg/ml of methylene blue (Fisher Scientific; catalog no.: M-291) in 2% dihydrate sodium citrate solution for 5 min at room temperature. Viable cells (those whose cell wall was stained blue) and metabolically active cells (those that excluded dye) were counted by light microscopy using a hemocytometer.

To quantify long-term viability, midlog RMY005 cells were diluted 2500-fold with YPDA following the aforementioned HS/HD treatment, and 50 μl (∼200–300 cells) were plated on rich medium. The cells were allowed to grow for 1.5 to 2 days before counting colony-forming units. Controls for this and the aforementioned assays were cells maintained at 25 °C throughout the HD/HS protocol.

## Data availability

All data described here are contained within the article as either a main figure or a supporting figure. Primary data will be made available upon request. Address all inquiries to David S. Gross (david.gross@lsuhs.edu).

## Supporting information

This article contains supporting information.

## Conflict of interest

The authors declare that they have no conflicts of interest with the contents of this article.
