# Supplementary material for: Phase-separation antagonists potently inhibit transcription and broadly increase nucleosome density
Source: J Biol Chem. 2022 Aug 11;298(10):102365. doi: 10.1016/j.jbc.2022.102365 (PMC9486037; doi:10.1016/j.jbc.2022.102365)
Supplement: Supplemental Tables S1–S2 [file mmc1.docx]

Supplemental Material

Table S1. Yeast Strains Used

| **Strain** | **Genotype** | **Source** |
| --- | --- | --- |
| W303-1A | *MATa ade2-1 trp1-1 can1-100 leu2-3,112 his3-11,15 ura3-1* | Rodney Rothstein |
| DPY304 | *W303-1A; ADE2 hsf1∆::KAN-MX; HSEv2-Venus::LEU2; HSF1::TRP1* | David Pincus |
| RMY005 | *DPY304; MEX67-3FV5::caURA3* | This study |
| LRY070 | *W303-1A; RPO31-MYCx9::TRP1* | This study |
| DBY1447 | *MATa/MATα LacIGFP:LEU2/leu2-3 trp1-1/trp1-1 can1-100/can1-100 p6LacO-SSA2/p6LacO-SSA4 ade2-1/ade2-1 LacIGFP:HIS3/his3-1 SEC63- MYCx13:KanMX HTA2-mCherry:spHIS5* | Donna and Jason Brickner |

**Table S2. Primers Used**

**RT-qPCR**

| **Primer** | **Sequence** |
| --- | --- |
| SCR1 F +385 | 5’ CGGCCGGGATAGCACATATC 3’ |
| SCR1 R +438 | 5’ CGCCGAAGCGATCAACTTG 3’ |
| HSP12 ORF F +9 | 5' CGCAGGTAGAAAAGGATTCG 3' |
| HSP12 ORF R +133 | 5' GAACCTTACCAGCGACCTTG 3' |
| HSP104 ORF F+1646 | 5' CAGCTGCAAGATTGACTGGTATCC 3' |
| HSP104 ORF R +1799 | 5' CCTGATCTAGACAATCTAACGGC 3' |
| HSP82 3' end ORF F | 5' GCTCCGGTTGAAGAGGTCCA 3' |
| HSP82 3' UTR R | 5' CTATTCAAGGCATGATGTTCTAC 3' |
| SSA4 ORF F +816 | 5' GTCTTCGTCTGCTCAGACATC 3' |
| SSA4 ORF R +946 | 5' CCACTGGCTCCAATGTAGATC 3' |
| CTT1 ORF F +193 | 5' GCCAAAGGTGGTGGTTGTAG 3' |
| CTT1 ORF R +314 | 5' ACGGTGGAAAAACGAACAAG 3' |
| PGM2 ORF F +914 | 5' TCGTTTCTCCAGGTGACTCC 3' |
| PGM2 ORF R +1038 | 5' AACACGGTCTATGGCTCCTG 3' |
| ACT1-intron-F | 5’ GTCTCATGTACTAACATCGATTGC 3’ |
| ACT1-exon2-R | 5’ CCGTTATCAATAACCAAAGCAGC 3’ |
| TUB1-5’UTR F | 5’ CCGTCTACAACAGTTCTCGCCAC 3’ |
| TUB1-intron R | 5’ CTGGACGGGCAAATCGAACATAC 3’ |

**ChIP-qPCR**

| **Primer** | **Sequence** |
| --- | --- |
| TUB1 5' UTR F | 5’ CCGTCTACAACAGTTCTCGCCAC 3’ |
| TUB1 intron R | 5’ CTGGACGGGCAAATCGAACATAC 3’ |
| TUB1 ORF F +703 | 5’ GAACATGCAGATTGTACTTTCATGGTC 3’ |
| TUB1 ORF R +789 | 5’ AGCTTGGTCTTGGGATATCCAAG 3’ |
| TUB1 3'UTR F +1406 | 5’ AAGAGATTACATCGAAGTGGGTGC 3’ |
| TUB1 3'UTR R +1488 | 5’ ATAAGGAGGTTGGGGGCGAGAG 3’ |
| ACT1 Prom F -192 | 5’ ATATCACGCTCTCTTTTTATCTTC 3’ |
| ACT1 Prom R -103 | 5’ CTTTTCTTTTTCTTCTTGGTTTGAG 3’ |
| ACT1 ORF F +811 | 5' GGTTTCTCTCTACCTCACGC 3' |
| ACT1 ORF R +879 | 5' CATCAAGTAGTCAGTCAAATCTCTACC 3' |
| ACT1 3'UTR F +1417 | 5’ GTTCACCACAAGTGTTTCTAATC 3’ |
| ACT1 3'UTR R +1510 | 5’ ATTGAGAGGGTGGTTTAAAAATAG 3’ |
| HSP104 UAS F -267 | 5' CTTAAACGTTCCATAAGGGGC 3' |
| HSP104 UAS R -196 | 5' TGCAGTTCTTTGAGATGGGCC 3' |
| HSP104 Prom F -130 | 5' GCATTGTAATCTTGCCTCAATTCC 3' |
| HSP104 Prom R -70 | 5' GTTATTGCTGATTCGATTCAAGG 3' |
| HSP104 ORF F +1469 | 5' CCCTTGATGCTGAACGTAGATATG 3' |
| HSP104 ORF R +1621 | 5' CCACATTTTGGATCATGGAGTTG 3' |
| HSP104 3'UTR F +2676 | 5' AGGTGATGACGATAATGAGGACAG 3' |
| HSP104 3'UTR R +2839 | 5' TCTTTTGCTCGGGTGTCAAGTTC 3' |
| PGM2 Prom F -225 | 5' GGAACAAACAGGGGTTGAAAAGTG 3' |
| PGM2 Prom R -60 | 5' ATCCTATTGTTCAAGCAACGCTG 3' |
| PGM2 ORF F +914 | 5' TCGTTTCTCCAGGTGACTCC 3' |
| PGM2 ORF R +1038 | 5' AACACGGTCTATGGCTCCTG 3' |
| PGM2 3' UTR F +1644 | 5' CTCGGTCATCAAGTTCTTGAAC 3' |
| PGM2 3'UTR R +1737 | 5' CATTAAGCCATTAGTAAATCATTCG 3' |
| HSP82 UAS F -393 | 5' CCTCTCTCAACACAGTAATCCATAAAC 3' |
| HSP82 UAS R -238 | 5' CTTCCACGGCGTTCTAGAAAAAAAAG 3' |
| HSP82 Prom F -157 | 5' TCCGCC ACCCCCTAAAAC 3' |
| HSP82 Prom R -113 | 5' TGAGGAGGTCACAGATGTTAAGAATT 3' |
| HSP82 ORF F +1248 | 5' GTTCTACTCGGCTTTCTCCAAAAATAT 3' |
| HSP82 ORF R +1444 | 5' CAGCCTTTAGAGATTCACCAGTGATG 3' |
| HSP82 3'UTR F +2134 | 5' AACATCATGGCCTTGAATAGGTTAT 3' |
| HSP82 3'UTR R +2228 | 5' CATGCAGATGCCCTATTTACATACTT 3' |
| HSP12 HSE -965F | 5' GTCCAGGTGGAGTGCGATTTGTTC 3' |
| HSP12 HSE -700R | 5' CCTACCTTCTCCCACTTTTCTGTG 3' |
| HSP12 ORF F +9 | 5' CGCAGGTAGAAAAGGATTCG 3' |
| HSP12 ORF R +133 | 5' GAACCTTACCAGCGACCTTG 3' |
| SSA4 UAS F -374 | 5' GCCGCACATCCATTCCGGTATG 3' |
| SSA4 UAS R -291 | 5' CGGGCAAAAGATATCCGCTTTG 3' |
| SSA4 Prom F -246 | 5' AGTTCCTAGAACCTTATGGAAGCAC3' |
| SSA4 Prom R +35 | 5' GTTGTACCTAAATCAATACCAACAGC 3' |
| SSA4 ORF F +816 | 5' GTCTTCGTCTGCTCAGACATC 3' |
| SSA4 ORF R +946 | 5' CCACTGGCTCCAATGTAGATC 3' |
| SSA4 3'UTR F +1762 | 5' GAGGAATACAAGGAAAGGCAAAAG 3' |
| SSA4 3'UTR R +2079 | 5' TTAAACTCTGGCTTATGACGATGAG 3' |
| HMLα F +339 | 5' TGTCTTGTCTTCTCTGCTCG 3' |
| HMLα R +473 | 5' GCATAATTATTCGTCAACCACTCTAC 3' |
| PHO5 Prom F -174 | 5' GGTCACCTTACTTGGCAAGGCA 3' |
| PHO5 Prom R -61 | 5' CCATACTAACCTCGACTTAGC 3' |
| RPR1 ORF +150 F | 5' CCTGCTCCTGAGAGAAGAAATATACTG 3' |
| RPR1 ORF +317 R | 5' GATTGCACTCAACAGACCTTGAC 3' |
| SCR1 F +385 | 5’ CGGCCGGGATAGCACATATC 3’ |
| SCR1 R +438 | 5’ CGCCGAAGCGATCAACTTG 3’ |
| YFR057W F +357 | 5' GCATACATATGAATATTACAACCAC 3' |
| YFR057W R +459 | 5' GCATTATGGCTTTGTTACG 3' |
| ARS504 F | 5' GTCAGACCTGTTCCTTTAAGAGG 3' |
| ARS504 R | 5' CATACCCTCGGGTCAAACAC 3' |
