# Supplementary material for: Phase-separation antagonists potently inhibit transcription and broadly increase nucleosome density
Source: J Biol Chem. 2022 Aug 11;298(10):102365. doi: 10.1016/j.jbc.2022.102365 (PMC9486037; doi:10.1016/j.jbc.2022.102365)
Supplement: Supplemental Figures S1–S4 [file mmc2.pptx]

## Slide 1
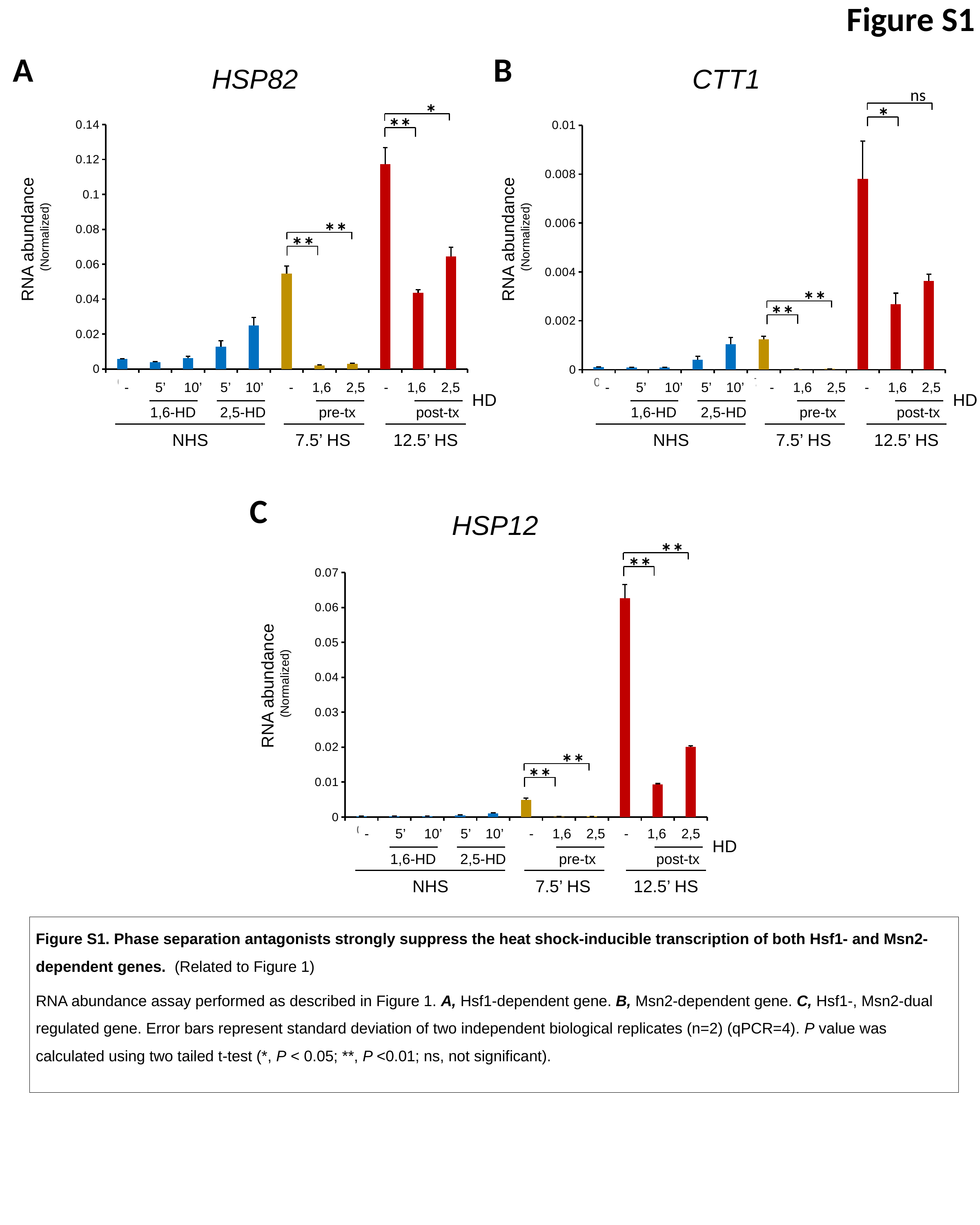

Figure S1
A
B
HSP82
CTT1
ns
*
### Chart
| Category | Relative to SCR1 |
|---|---|
| 0' | 0.005762852852734162 |
| 5' | 0.0038333696774694925 |
| 10' | 0.006183743423539292 |
| 5' | 0.012908988828919145 |
| 10' | 0.024938784036334764 |
| 7.5' | 0.05473395171001758 |
| 7.5' | 0.0020316394385561844 |
| 7.5' | 0.0029685633257969897 |
| 12.5' | 0.11739507430247628 |
| 12.5' | 0.04369251919480403 |
| 12.5' | 0.06449996364247293 |
### Chart
| Category | Relative to SCR1 |
|---|---|
| 0' | 0.00010050596934838722 |
| 5' | 9.388935813868336e-05 |
| 10' | 9.552774115901934e-05 |
| 5' | 0.000403134315684938 |
| 10' | 0.0010455240108142496 |
| 7.5' | 0.0012395233771677508 |
| 7.5' | 1.9606092547606245e-05 |
| 7.5' | 3.291414240351284e-05 |
| 12.5' | 0.007803491633246515 |
| 12.5' | 0.0026851207623527704 |
| 12.5' | 0.0036308301940159975 |*
**
**
**
RNA abundance
(Normalized)
RNA abundance
(Normalized)
**
**
- 5’ 10’ 5’ 10’
 - 1,6 2,5 - 1,6 2,5
- 5’ 10’ 5’ 10’
 - 1,6 2,5 - 1,6 2,5
HD
HD
1,6-HD
2,5-HD
pre-tx
post-tx
1,6-HD
2,5-HD
pre-tx
post-tx
NHS
7.5’ HS
12.5’ HS
NHS
7.5’ HS
12.5’ HS
C
HSP12
### Chart
| Category | Relative to SCR1 |
|---|---|
| 0' | 0.00020980194995686325 |
| 5' | 0.000244729896565482 |
| 10' | 0.00023889678105255103 |
| 5' | 0.0004521534169950274 |
| 10' | 0.0010556156606419333 |
| 7.5' | 0.004882000356949837 |
| 7.5' | 9.573017600428399e-05 |
| 7.5' | 0.00016660081976390575 |
| 12.5' | 0.06267475629282367 |
| 12.5' | 0.009290289203357085 |
| 12.5' | 0.02012649227048197 |**
**
RNA abundance
(Normalized)
**
**
- 5’ 10’ 5’ 10’
 - 1,6 2,5 - 1,6 2,5
HD
1,6-HD
2,5-HD
pre-tx
post-tx
NHS
7.5’ HS
12.5’ HS
Figure S1. Phase separation antagonists strongly suppress the heat shock-inducible transcription of both Hsf1- and Msn2-dependent genes. (Related to Figure 1)
RNA abundance assay performed as described in Figure 1. A, Hsf1-dependent gene. B, Msn2-dependent gene. C, Hsf1-, Msn2-dual regulated gene. Error bars represent standard deviation of two independent biological replicates (n=2) (qPCR=4). P value was calculated using two tailed t-test (*, P < 0.05; **, P <0.01; ns, not significant).

## Slide 2
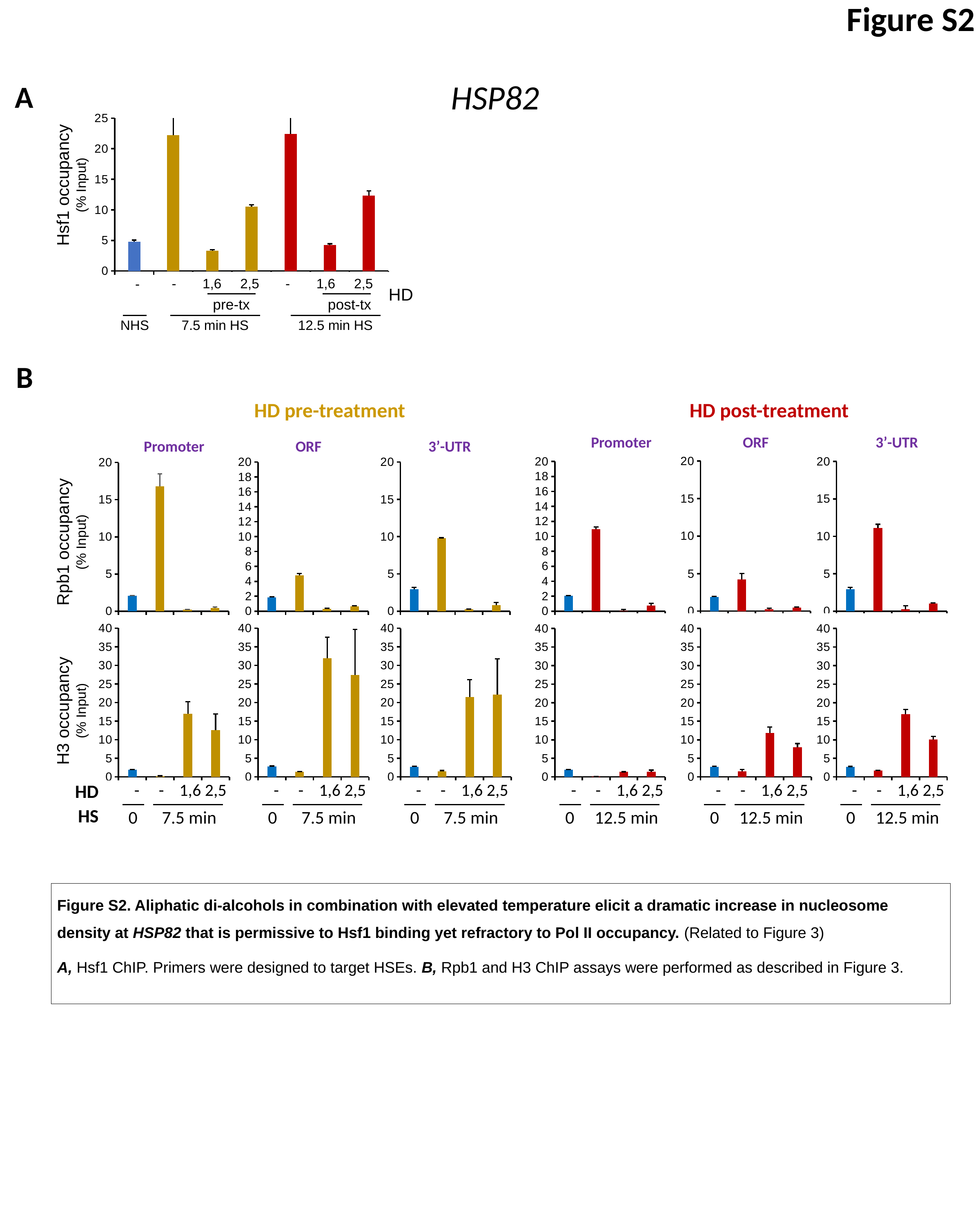

Figure S2
HSP82
A
### Chart
| Category | Percentage of Input |
|---|---|
| 0' | 4.776856359234197 |
| 7.5' | 22.234308483864886 |
| 7.5' | 3.3377447117930297 |
| 7.5' | 10.557336093643464 |
| 12.5' | 22.453378208544397 |
| 12.5' | 4.249704774348485 |
| 12.5' | 12.338751497004964 |Hsf1 occupancy
(% Input)
 - 1,6 2,5 - 1,6 2,5
-
HD
pre-tx
post-tx
NHS
7.5 min HS
12.5 min HS
B
HD pre-treatment
HD post-treatment
Promoter
ORF
3’-UTR
Promoter
ORF
3’-UTR
### Chart
| Category | Percentage of Input |
|---|---|
| 0' | 1.8675432458199022 |
| 12.5' | 4.218109951900818 |
| 12.5' | 0.26805883744267317 |
| 12.5' | 0.4760047918521358 |
### Chart
| Category | Percentage of Input |
|---|---|
| 0' | 2.0754843552292757 |
| 12.5' | 10.932948423084827 |
| 12.5' | 0.030831607053837327 |
| 12.5' | 0.7459322420557895 |
### Chart
| Category | Percentage of Input |
|---|---|
| 0' | 2.9470546237682873 |
| 12.5' | 11.090851760263506 |
| 12.5' | 0.2467491774441527 |
| 12.5' | 0.9997383787820854 |
### Chart
| Category | Percentage of Input |
|---|---|
| 0' | 1.8675432458199022 |
| 7.5' | 4.790579711288704 |
| 7.5' | 0.32822959721657957 |
| 7.5' | 0.6616592609067549 |
### Chart
| Category | Percentage of Input |
|---|---|
| 0' | 2.9470546237682873 |
| 7.5' | 9.811734954816282 |
| 7.5' | 0.24614095043871076 |
| 7.5' | 0.8334974330293243 |
### Chart
| Category | Percentage of Input |
|---|---|
| 0' | 2.0754843552292757 |
| 7.5' | 16.809587112846092 |
| 7.5' | 0.20345279888534973 |
| 7.5' | 0.43524025046982034 |Rpb1 occupancy
(% Input)
### Chart
| Category | Percentage of Input |
|---|---|
| 0' | 1.9426946171347597 |
| 7.5' | 0.20308608664160416 |
| 7.5' | 16.939983947399206 |
| 7.5' | 12.620668203620149 |
### Chart
| Category | Percentage of Input |
|---|---|
| 0' | 2.7584326406994464 |
| 7.5' | 1.4132940567764252 |
| 7.5' | 31.92309296905208 |
| 7.5' | 27.396503560522937 |
### Chart
| Category | Percentage of Input |
|---|---|
| 0' | 2.7099942678660476 |
| 7.5' | 1.4985720365032484 |
| 7.5' | 21.513805834846707 |
| 7.5' | 22.201116490879784 |
### Chart
| Category | Percentage of Input |
|---|---|
| 0' | 1.9426946171347597 |
| 12.5' | 0.10900646110056839 |
| 12.5' | 1.3509376797068486 |
| 12.5' | 1.3575147659994173 |
### Chart
| Category | Percentage of Input |
|---|---|
| 0' | 2.7584326406994464 |
| 12.5' | 1.4787108666708366 |
| 12.5' | 11.825162989298246 |
| 12.5' | 7.94880279962913 |
### Chart
| Category | Percentage of Input |
|---|---|
| 0' | 2.7099942678660476 |
| 12.5' | 1.666084626020802 |
| 12.5' | 16.889317734991018 |
| 12.5' | 10.047014835440322 |H3 occupancy
(% Input)
- - 1,6 2,5
0 7.5 min
- - 1,6 2,5
0 7.5 min
- - 1,6 2,5
0 7.5 min
- - 1,6 2,5
0 12.5 min
- - 1,6 2,5
0 12.5 min
- - 1,6 2,5
0 12.5 min
HD
HS
Figure S2. Aliphatic di-alcohols in combination with elevated temperature elicit a dramatic increase in nucleosome density at HSP82 that is permissive to Hsf1 binding yet refractory to Pol II occupancy. (Related to Figure 3)
A, Hsf1 ChIP. Primers were designed to target HSEs. B, Rpb1 and H3 ChIP assays were performed as described in Figure 3.

## Slide 3
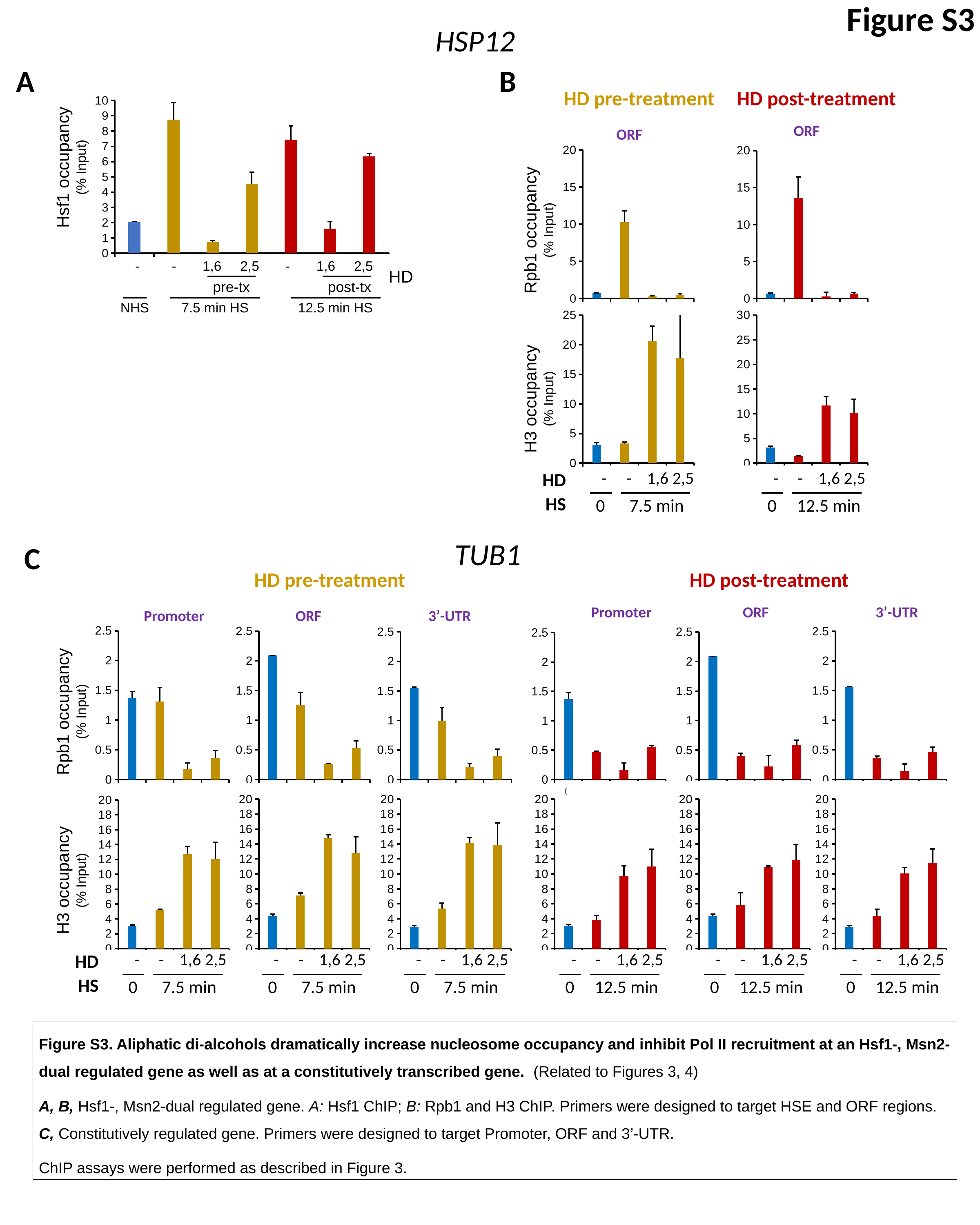

Figure S3
HSP12
A
B
HD pre-treatment
HD post-treatment
### Chart
| Category | Percentage of Input |
|---|---|
| 0' | 2.0406781886952157 |
| 7.5' | 8.74078114266889 |
| 7.5' | 0.7659579388752955 |
| 7.5' | 4.533713488140848 |
| 12.5' | 7.433181268723496 |
| 12.5' | 1.6112917976076537 |
| 12.5' | 6.344916385171478 |ORF
ORF
### Chart
| Category | Percentage of Input |
|---|---|
| 0' | 0.6891437588905007 |
| 7.5' | 10.309176654058778 |
| 7.5' | 0.28814993534750305 |
| 7.5' | 0.47696975801024105 |
### Chart
| Category | Percentage of Input |
|---|---|
| 0' | 0.6891437588905007 |
| 12.5' | 13.579685700948112 |
| 12.5' | 0.28976570436487475 |
| 12.5' | 0.6382095996042509 |Hsf1 occupancy
(% Input)
Rpb1 occupancy
(% Input)
 - 1,6 2,5 - 1,6 2,5
-
HD
pre-tx
post-tx
NHS
7.5 min HS
12.5 min HS
### Chart
| Category | Percentage of Input |
|---|---|
| 0' | 3.125195099150583 |
| 7.5' | 3.288596862927604 |
| 7.5' | 20.593939142517435 |
| 7.5' | 17.807501375351915 |
### Chart
| Category | Percentage of Input |
|---|---|
| 0' | 3.125195099150583 |
| 12.5' | 1.4197791454860593 |
| 12.5' | 11.693725913971129 |
| 12.5' | 10.209997729675933 |
H3 occupancy
(% Input)
- - 1,6 2,5
0 7.5 min
- - 1,6 2,5
0 12.5 min
HD
HS
TUB1
C
HD pre-treatment
HD post-treatment
Promoter
ORF
3’-UTR
Promoter
ORF
3’-UTR
### Chart
| Category | Percentage of Input |
|---|---|
| 0' | 1.369188860499177 |
| 7.5' | 1.3107987279572177 |
| 7.5' | 0.17669185138793145 |
| 7.5' | 0.3618454003548592 |
### Chart
| Category | Percentage of Input |
|---|---|
| 0' | 2.0894649721055623 |
| 7.5' | 1.2565514869831245 |
| 7.5' | 0.2616685939014053 |
| 7.5' | 0.5336847269782682 |
### Chart
| Category | Percentage of Input |
|---|---|
| 0' | 1.5548053220659808 |
| 12.5' | 0.36814646969345366 |
| 12.5' | 0.14694373519983184 |
| 12.5' | 0.46955004040421267 |
### Chart
| Category | Percentage of Input |
|---|---|
| 0' | 1.5548053220659808 |
| 7.5' | 0.9889454503337949 |
| 7.5' | 0.21629675901312928 |
| 7.5' | 0.3969152409632609 |
### Chart
| Category | Percentage of Input |
|---|---|
| 0' | 2.0894649721055623 |
| 12.5' | 0.3992731189043949 |
| 12.5' | 0.2185113193461493 |
| 12.5' | 0.5796357580502249 |
### Chart
| Category | Percentage of Input |
|---|---|
| 0' | 1.369188860499177 |
| 12.5' | 0.4722419404192821 |
| 12.5' | 0.16766098476088265 |
| 12.5' | 0.5504908928481322 |Rpb1 occupancy
(% Input)
### Chart
| Category | Percentage of Input |
|---|---|
| 0' | 4.340723255269868 |
| 7.5' | 7.099386641478475 |
| 7.5' | 14.839850668282237 |
| 7.5' | 12.813097828551628 |
### Chart
| Category | Percentage of Input |
|---|---|
| 0' | 2.89409159902405 |
| 7.5' | 5.344816425057876 |
| 7.5' | 14.1719935820165 |
| 7.5' | 13.895675365553432 |
### Chart
| Category | Percentage of Input |
|---|---|
| 0' | 3.0407525083462925 |
| 12.5' | 3.8388497786920777 |
| 12.5' | 9.66443160191641 |
| 12.5' | 10.97645447969727 |
### Chart
| Category | Percentage of Input |
|---|---|
| 0' | 4.340723255269868 |
| 12.5' | 5.864396920643287 |
| 12.5' | 10.901661482062224 |
| 12.5' | 11.871956776304367 |
### Chart
| Category | Percentage of Input |
|---|---|
| 0' | 2.89409159902405 |
| 12.5' | 4.350106735074431 |
| 12.5' | 10.040896133933185 |
| 12.5' | 11.503695570266167 |
### Chart
| Category | Percentage of Input |
|---|---|
| 0' | 3.0407525083462925 |
| 7.5' | 5.223200971848968 |
| 7.5' | 12.68584845170365 |
| 7.5' | 12.051891098248412 |H3 occupancy
(% Input)
- - 1,6 2,5
0 7.5 min
- - 1,6 2,5
0 7.5 min
- - 1,6 2,5
0 7.5 min
- - 1,6 2,5
0 12.5 min
- - 1,6 2,5
0 12.5 min
- - 1,6 2,5
0 12.5 min
HD
HS
Figure S3. Aliphatic di-alcohols dramatically increase nucleosome occupancy and inhibit Pol II recruitment at an Hsf1-, Msn2-dual regulated gene as well as at a constitutively transcribed gene. (Related to Figures 3, 4)
A, B, Hsf1-, Msn2-dual regulated gene. A: Hsf1 ChIP; B: Rpb1 and H3 ChIP. Primers were designed to target HSE and ORF regions. C, Constitutively regulated gene. Primers were designed to target Promoter, ORF and 3’-UTR.
ChIP assays were performed as described in Figure 3.

## Slide 4
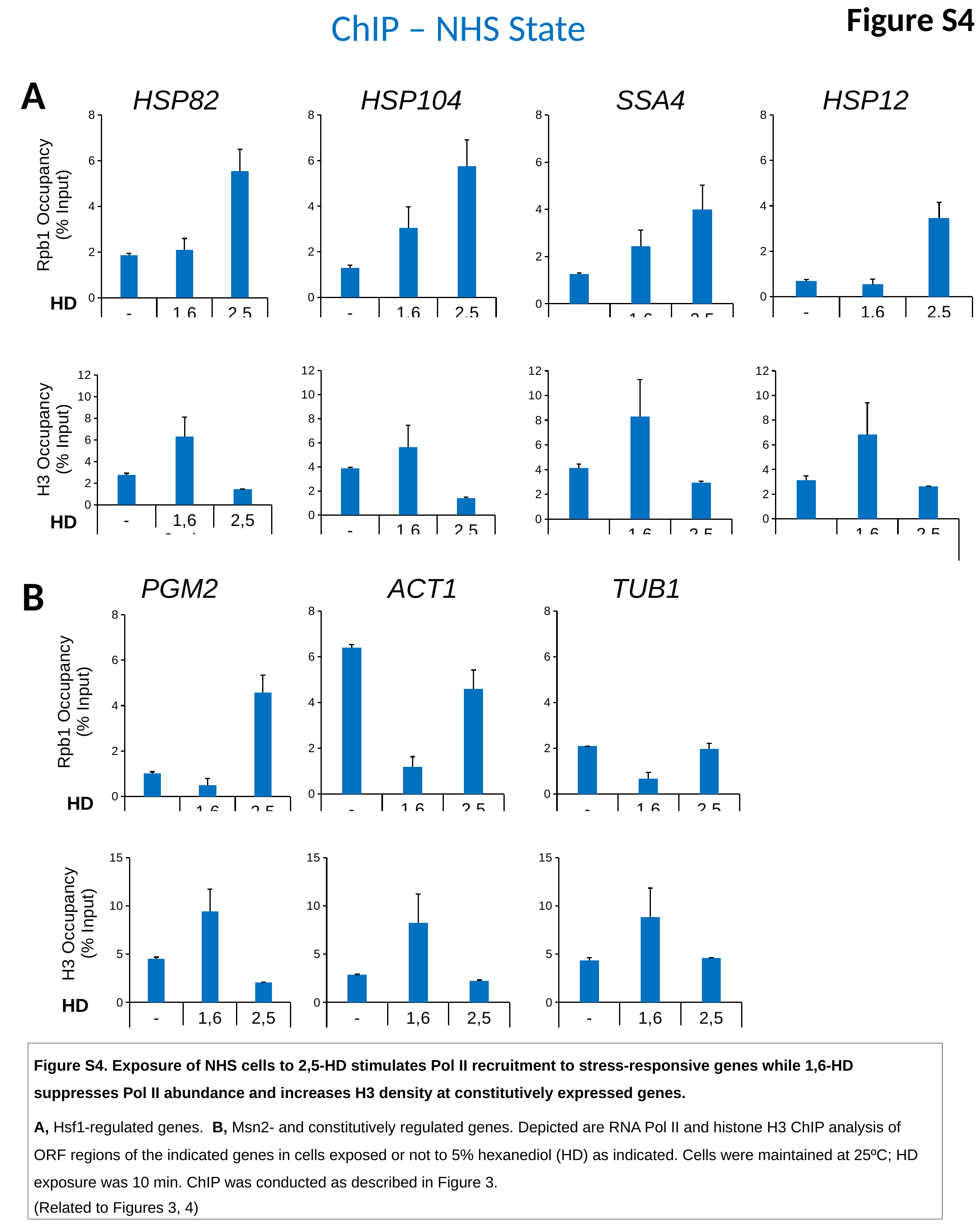

Figure S4
ChIP – NHS State
A
HSP82
HSP104
SSA4
HSP12
### Chart
| Category | Percentage of Input |
|---|---|
| - | 1.8675432458199022 |
| 1,6 | 2.0917059000289617 |
| 2,5 | 5.536404327084908 |
### Chart
| Category | Percentage of Input |
|---|---|
| - | 1.2912889703041017 |
| 1,6 | 3.0519428246603457 |
| 2,5 | 5.7485907133679275 |
### Chart
| Category | Percentage of Input |
|---|---|
| - | 1.2652445336089548 |
| 1,6 | 2.4366408000861925 |
| 2,5 | 4.005061849984573 |
### Chart
| Category | Percentage of Input |
|---|---|
| - | 0.6891437588905007 |
| 1,6 | 0.5424344112817602 |
| 2,5 | 3.4564719274935767 |HD
HS
### Chart
| Category | Percentage of Input |
|---|---|
| - | 2.7584326406994464 |
| 1,6 | 6.322105342111283 |
| 2,5 | 1.4288676944888132 |
### Chart
| Category | Percentage of Input |
|---|---|
| - | 3.891372609616572 |
| 1,6 | 5.644029179820391 |
| 2,5 | 1.4196103718483553 |
### Chart
| Category | Percentage of Input |
|---|---|
| - | 4.149507230457285 |
| 1,6 | 8.303777743195521 |
| 2,5 | 2.9570425896879566 |
### Chart
| Category | Percentage of Input |
|---|---|
| - | 3.125195099150583 |
| 1,6 | 6.8411114155955595 |
| 2,5 | 2.6301666550035225 |HD
HS
B
PGM2
ACT1
TUB1
### Chart
| Category | Percentage of Input |
|---|---|
| - | 6.405769480697162 |
| 1,6 | 1.1914858135318647 |
| 2,5 | 4.597769641471842 |
### Chart
| Category | Percentage of Input |
|---|---|
| - | 2.0894649721055623 |
| 1,6 | 0.6650862922857947 |
| 2,5 | 1.971392692410046 |
### Chart
| Category | Percentage of Input |
|---|---|
| - | 1.006600025847019 |
| 1,6 | 0.5025838699640515 |
| 2,5 | 4.575947406962053 |HD
HS
### Chart
| Category | Percentage of Input |
|---|---|
| - | 2.8614171502853862 |
| 1,6 | 8.236512629434971 |
| 2,5 | 2.247523053647104 |
### Chart
| Category | Percentage of Input |
|---|---|
| - | 4.340723255269868 |
| 1,6 | 8.849109691858532 |
| 2,5 | 4.598266822888965 |
### Chart
| Category | Percentage of Input |
|---|---|
| - | 4.5067778029011 |
| 1,6 | 9.416593756622834 |
| 2,5 | 2.0508670545458596 |HD
HS
Figure S4. Exposure of NHS cells to 2,5-HD stimulates Pol II recruitment to stress-responsive genes while 1,6-HD suppresses Pol II abundance and increases H3 density at constitutively expressed genes.
A, Hsf1-regulated genes. B, Msn2- and constitutively regulated genes. Depicted are RNA Pol II and histone H3 ChIP analysis of ORF regions of the indicated genes in cells exposed or not to 5% hexanediol (HD) as indicated. Cells were maintained at 25ºC; HD exposure was 10 min. ChIP was conducted as described in Figure 3.
(Related to Figures 3, 4)
